# The *TSC2* c.2742+5G>A variant causes variable splicing changes and clinical manifestations in a family with tuberous sclerosis complex

**DOI:** 10.3389/fnmol.2023.1091323

**Published:** 2023-04-20

**Authors:** Kuan Fan, Yi Guo, Zhi Song, Lamei Yuan, Wen Zheng, Xiao Hu, Lina Gong, Hao Deng

**Affiliations:** ^1^Department of Health Management, The Third Xiangya Hospital, Central South University, Changsha, China; ^2^Center for Experimental Medicine, The Third Xiangya Hospital, Central South University, Changsha, China; ^3^Department of Neurology, Guizhou Provincial People's Hospital, Guiyang, China; ^4^Department of Neurology, The Third Xiangya Hospital, Central South University, Changsha, China

**Keywords:** tuberous sclerosis complex, *TSC2* gene, splicing variant, non-canonical splice site, alternative splicing

## Abstract

**Background:**

Tuberous sclerosis complex (TSC) is a genetic, variably expressed, multisystem disease characterized by benign tumors. It is caused by pathogenic variants of the TSC complex subunit 1 gene (*TSC1*) and the TSC complex subunit 2 gene (*TSC2*). Genetic testing allows for early diagnosis, genetic counseling, and improved outcomes, but it did not identify a pathogenic variant in up to 25% of all TSC patients. This study aimed to identify the disease-causing variant in a Han-Chinese family with TSC.

**Methods:**

A six-member, three-generation Han-Chinese family with TSC and three unrelated healthy women were recruited. A comprehensive medical examination, a 3-year follow-up, whole exome sequencing, Sanger sequencing, and segregation analysis were performed in the family. The splicing analysis results obtained from six *in silico* tools, minigene assay, and patients' lymphocyte messenger RNA were compared, and quantitative reverse transcription PCR was used to confirm the pathogenicity of the variant.

**Results:**

Two affected family members had variable clinical manifestations including a rare bilateral cerebellar ataxia symptom. The 3-year follow-up results suggest the effects of a combined treatment of anti-epilepsy drugs and sirolimus for TSC-related epilepsy and cognitive deficits. Whole exome sequencing, Sanger sequencing, segregation analysis, splicing analysis, and quantitative reverse transcription PCR identified the *TSC2* gene c.2742+5G>A variant as the genetic cause. This variant inactivated the donor splice site, a cryptic non-canonical splice site was used for different splicing changes in two affected subjects, and the resulting mutant messenger RNA may be degraded by nonsense-mediated decay. The defects of *in silico* tools and minigene assay in predicting cryptic splice sites were suggested.

**Conclusions:**

This study identified a *TSC2* c.2742+5G>A variant as the genetic cause of a Han-Chinese family with TSC and first confirmed its pathogenicity. These findings expand the phenotypic and genetic spectrum of TSC and may contribute to its diagnosis and treatment, as well as a better understanding of the splicing mechanism.

## 1. Introduction

Tuberous sclerosis complex (TSC) is an autosomal dominant disorder characterized by histologically benign lesions in multiple organs and systems (Curatolo et al., 2015; Henske et al., 2016). Globally, it affected almost 2,000,000 people, and the incidence rate varies between 4.47 × 10^−5^ and 1.72 × 10^−4^ among newborns (Osborne et al., 1991; Henske et al., 2016; Ebrahimi-Fakhari et al., 2018). This disease is caused by pathogenic variants of two genes: the TSC complex subunit 1 gene (*TSC1*, OMIM 605284) contains 23 exons on chromosome 9q34.13, and the TSC complex subunit 2 gene (*TSC2*, OMIM 191092) contains 42 exons on chromosome 16p13.3 (Roach, 2016). These two genes encode hamartin (also known as TSC1) and tuberin (also known as TSC2) proteins (Curatolo et al., 2015). They form an intracellular heterotrimeric complex to regulate the activity of the mechanistic target of rapamycin (mTOR) complex 1 *via* stimulating the GTP hydrolysis of the RAS homolog enriched in brain protein (Inoki et al., 2003). *TSC1* or *TSC2* pathogenic variants over-activate mTOR-mediated signaling with consequent extensive metabolic reprogramming (Henske et al., 2016). This results in uncontrolled cell growth and proliferation and a nervous system imbalance between excitation and inhibition (Curatolo et al., 2015; Roach, 2016). These effects lead to various clinical manifestations including skin, brain, lungs, heart, and kidney tumors and neurological disorders, such as epilepsy, autism spectrum disorder (ASD), and attention deficit hyperactivity disorder (ADHD) (Henske et al., 2016; Roach, 2016; de Vries et al., 2018). There is great symptom variability among affected individuals, even identical twins (Humphrey et al., 2004; Roach, 2016). It complicates diagnosis, particularly in younger patients and those with milder symptoms (Northrup et al., 2013). The use of mTOR inhibitors enables early diagnosis and treatment to be an opportunity for better clinical outcomes (Chung et al., 2017; Ebrahimi-Fakhari et al., 2018). According to the TSC diagnostic criteria updated in 2012, detecting *TSC1* and *TSC2* genes pathogenic variants has been considered sufficient for diagnosis and facilitates early diagnosis (Northrup et al., 2013). Conventional genetic testing did not detect pathogenic variants in 10–25% of all TSC patients (Northrup et al., 2013). Identifying the pathogenicity of variants in introns possibly affecting splicing may partially explain the false negative rate (Tyburczy et al., 2015). In addition to determining the loss of wild-type splice sites, it is also necessary to find cryptic sites that may be used by the splicing mechanism because new splice sites close to the destroyed wild-type site possibly only lead to the deletion or insertion of a few amino acids (Houdayer et al., 2012). *In silico* prediction, minigene assay, and analyzing the messenger RNA (mRNA) extracted from patients are the main methods to identify variants that affect splicing. This study identified a *TSC2* intron variant (c.2742+5G>A), confirmed its pathogenicity in a family with TSC, and compared the splicing analyses results obtained from six prediction tools, minigene assay, and patients' lymphocyte mRNA. Different splicing changes in two affected subjects were caused by the use of a cryptic non-canonical alternative splice site. Patients' clinical presentations and a 3-year follow-up were reported. These findings have a potential value in TSC diagnosis and treatment, as well as a better understanding of the splicing mechanism.

## 2. Materials and methods

### 2.1. Subjects and clinical evaluations

**Figure 1:**
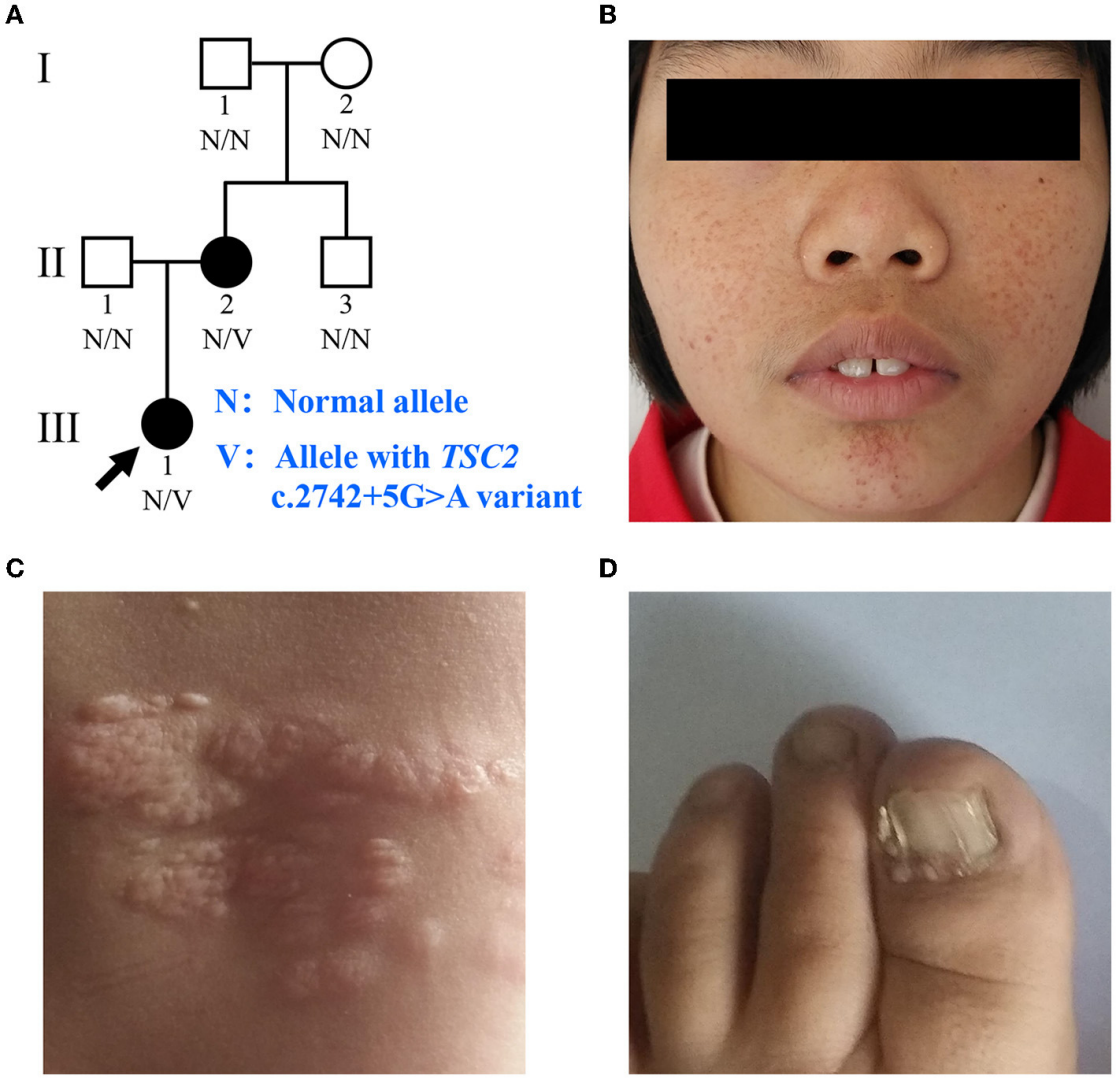
Clinical manifestations of the family with TSC. **(A)** Pedigree of the family with TSC. N, normal allele; V, allele with the *TSC2* c.2742+5G>A variant. The arrow indicates the proband. **(B)** Facial angiofibromas of the proband. **(C)** Shagreen patches of the proband. **(D)** Ungual fibromas of patient II:2.

A total of six members of a three-generation Han-Chinese family with TSC were recruited at the Third Xiangya Hospital, Central South University, China (Figure 1A). All of them were examined by experienced dermatologists, neurologists, and psychiatrists. TSC diagnosis was made according to the diagnostic criteria updated by the 2012 International Tuberous Sclerosis Complex Consensus Group (Northrup et al., 2013). The diagnostic and statistical manual of mental disorders (Fifth Edition) was used to make ADHD and ASD diagnoses (American Psychiatric Association, 2013). Physical examinations, head MRI, chest and abdomen CT, scanning laser ophthalmoscopy, electroencephalography (EEG), ultrasonic cardiogram, routine kidney function blood test, Mini-Mental State Examination (MMSE), Montreal Cognitive Assessment (MoCA), and Scale for the Assessment and Rating of Ataxia (SARA) were performed. Overall, three unrelated healthy Han-Chinese women (mean age 30.33 ± 2.62 years) were enrolled as controls. This study was approved by the Institutional Review Board of the Third Xiangya Hospital of Central South University. After written informed consent was obtained, peripheral venous blood samples were taken from each subject.

### 2.2. Variant analysis

Genomic DNA (gDNA) from peripheral venous blood lymphocytes was extracted using a standard phenol-chloroform extraction method. Whole exome sequencing (WES) was performed on the proband (III:1) by a commercial service from BGI-Shenzhen (Shenzhen, China) as previously described (Fan et al., 2019). In brief, gDNA samples were sonically fragmented into 150–250 bp. After end repair and adapter ligation, size-selected DNA fragments were amplified, purified, and then hybridized to the exome array for enrichment. Subsequently, high-throughput sequencing was performed to comprehensively search for candidate variants by the BGISEQ-500 sequencing platform. The clean data were mapped to the human reference genome sequence from the UCSC database (version hg19) using Burrows-Wheeler Alignment (version 0.7.15). Variants were detected by the Genome Analysis Toolkit (version 3.3.0) and annotated using ANNOVAR software. All variants were filtered to remove polymorphism according to the Single Nucleotide Polymorphism database (version 141), Genome Aggregation Database, and an in-house exome database of BGI. Coding insertions–deletions (indels), potential splice site changes, and non-synonymous single nucleotide variants (SNVs) in exons with a minor allele frequency of <10^−3^ were considered as candidates. Candidate variants in the *TSC1, TSC2*, and other infantile epilepsy-related genes were further predicted for pathogenicity and validated by Sanger sequencing. The RegRNA (version 2.0), NetGene2 (version 2.4), Splice Site Prediction by Neural Network (NNSPLICE, version 0.9), Alternative Splice Site Predictor (ASSP), Maximum Entropy Scan (MaxEntScan), and Human Splicing Finder (HSF, professional version) were used to evaluate the effects of variants on splicing signals and predict cryptic splice sites (Hebsgaard et al., 1996; Reese et al., 1997; Eng et al., 2004; Wang and Marín, 2006; Desmet et al., 2009; Chang et al., 2013). Sanger sequencing using an ABI3500 sequencer (Applied Biosystems Inc., Foster City, CA, USA) for detecting the potential disease-causing variant was performed in all subjects. Primer sequences for detecting *TSC2* c.2742+5G>A variant are shown in Supplementary Table 1. The gDNA samples of the proband and her immediate relatives were sequenced three times. A preliminary comparison of relative electropherogram peak heights for the variant allele was done to exclude mosaic variants using Chromas (version 1.62). Statistical analysis was performed using an independent sample student's *t*-test. All variants are described based on the reference sequences NG_005895.1, NM_000548.5, and NP_000539.2 in this study.

### 2.3. Minigene assay

The wild-type and mutant DNA sequences spanning *TSC2* gene exons 23–27 and introns 23–26 were amplified from the gDNA of the patient II:2 and introduced into the pMini-SP vector (Beijing Hitrobio Biotechnology Co., Ltd., Beijing, China) (Yuan et al., 2021). Human embryonic kidney (HEK) 293T cells were prepared in Dulbecco's modified Eagle's medium supplemented with 10% fetal bovine serum (HyClone, Logan, Utah, USA) at 37°C and 5% CO_2_. After Sanger sequencing validation, the wild-type and mutant plasmids were transfected into HEK293T cells using Lipofectamine 2000 (Thermo Fisher Scientific, Waltham, MA, USA). At 48 h post-transfection, total RNA was extracted from cells with TRIzol reagent (Thermo Fisher Scientific). Complementary DNA (cDNA) was reverse transcribed using a ReverTra Ace qPCR RT Master Mix with the gDNA Remover kit (TOYOBO Co., Ltd., Tokyo, Japan) from total RNA. The cDNA sequences of recombined plasmids were amplified by PCR. PCR products were analyzed through electrophoresis on a 1% agarose gel. Isoforms were purified using a Gel Extraction kit (Omega Bio-tek, Inc., Norcross, GA, USA) and determined by Sanger sequencing. To inhibit mRNA decay, cells transfected with wild-type and mutant minigene plasmids were incubated with dimethyl sulfoxide (DMSO) or cycloheximide (CHX, 100 μg/ml) for 24 h before total RNA isolation. Each total RNA was subdivided and reverse transcribed into three cDNA samples, and quantitative real-time PCR was performed in triplicate for each cDNA sample. Quantitative real-time PCR was performed on a LightCycler 480 Instrument II (Roche Molecular Systems, Inc., Indianapolis, IN, USA) with KOD SYBR qPCR Mix (TOYOBO Co., Ltd., Tokyo, Japan), and we optimized PCR conditions to analyze relative RNA levels of minigene sequences. The actin beta gene mRNA sequence was amplified as the endogenous control to estimate relative mRNA levels. Amplicon quality was verified by the melting curve analysis and agarose gel electrophoresis. Relative levels of mRNA were analyzed using the 2^−ΔΔCt^ method. One-way analysis of variance followed by the Bonferroni *post-hoc* test was used to determine the significance of differences among groups. Primer pairs used for minigene assay appear in Supplementary Table 1.

### 2.4. Lymphocyte mRNA analysis

Lymphocytes were separated from EDTA-anticoagulated blood samples of three family members (II:1, II:2, and III:1) and three controls using a human lymphocyte separation medium (Beijing Solarbio Science & Technology Co., Ltd., Beijing, China) in half an hour. After washing with PBS twice, TRIzol reagent was used to extract total RNA from lymphocytes. Reverse transcription PCR was performed to amplify cDNA sequences between exons 23 and 25 of the *TSC2* gene. PCR products were analyzed, separated, and determined as described above. Subsequently, primer pairs were designed to target wild-type and mutant *TSC2* mRNA sequences to analyze relative *TSC2* mRNA levels in total RNA extracted from patients' lymphocytes. The quantitative real-time PCR examination on lymphocyte RNA was performed and analyzed as described above. Primer pairs used in lymphocyte mRNA analysis are listed in Supplementary Table 1.

## 3. Results

### 3.1. Clinical presentations

**Figure 2:**
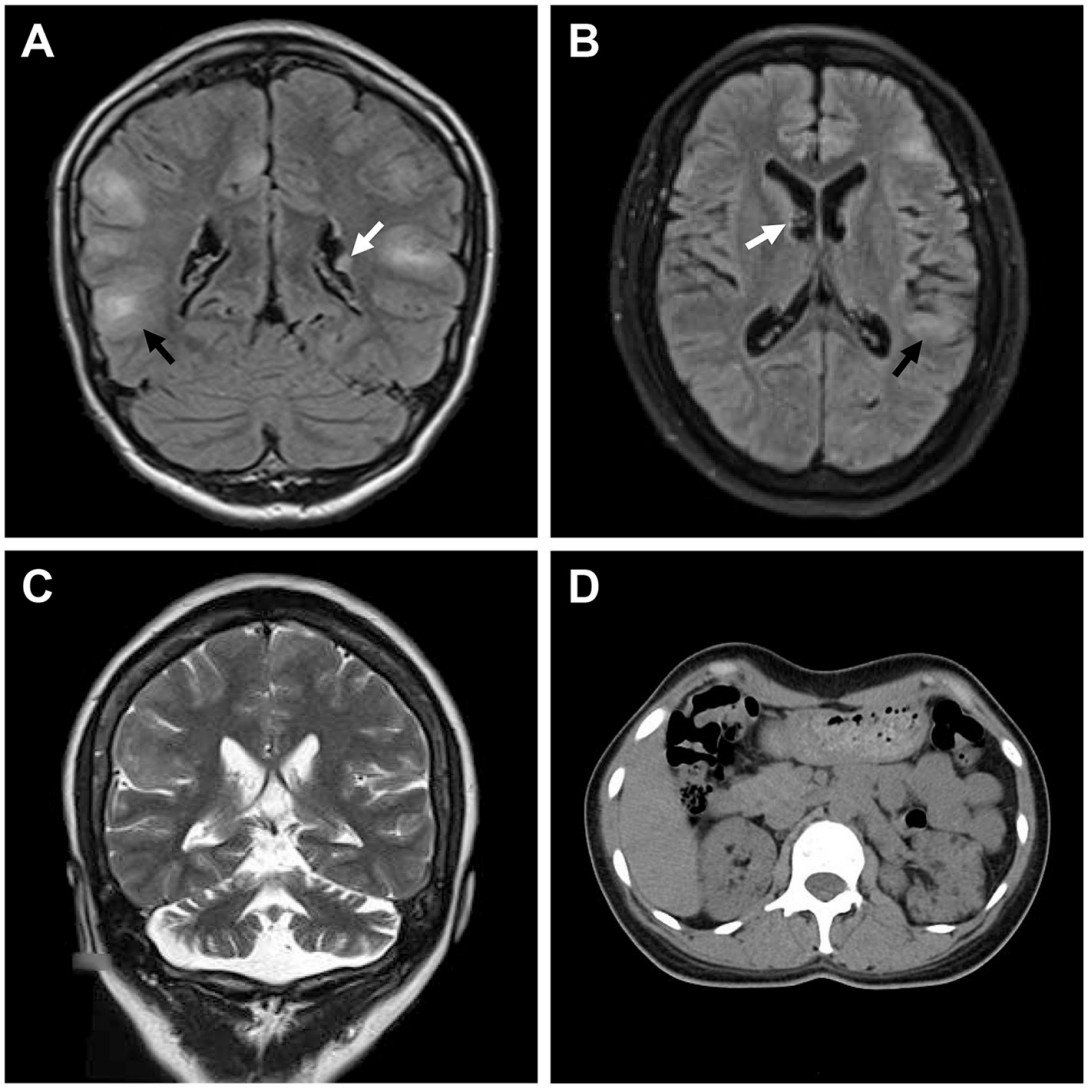
Medical images of patients with TSC. **(A)** Water-suppressed T2-weighted MRI sequence of the proband and **(B)** water and fat-suppressed T2-weighted MRI sequence of patient II:2, showing cortical dysplasia (black arrow) and subependymal nodules (white arrow). **(C)** T2-weighted MRI sequence of patient II:2, showing cerebellar atrophy. **(D)** CT scan of patient II:2, showing renal angiomyolipomas and cysts.

The proband (III:1), a 12-year-old female, was admitted to the hospital for drug-resistant epilepsy (Figure 1A). She presented with head nodding episodes that occur in clusters at 6 months of age. In the following years, seizure symptom intensity gradually increased despite various standard treatments, including adrenocorticotropic hormone, valproic acid, oxcarbazepine, and a ketogenic diet. Her condition had evolved into weekly tonic-clonic seizures lasting a few minutes. At 1 year of age, small swellings appeared on her cheeks, and irregularly thickened plaques appeared on her lower back. She had lower intelligence than her peers from the age of 3 years and manifested obvious inattention and learning disability. Physical examination found hypomelanotic macules, facial angiofibromas, fibrous plaques, shagreen patches, dental enamel pits, and intraoral fibromas (Figures 1B, C). ADHD was diagnosed, and no communication difficulty or ataxia symptoms were observed. Cranial MRI and CT scans detected cortical dysplasia and subependymal nodules (Figure 2A). EEG showed paroxysmal slow waves and epileptiform discharges in the right frontal and temporal lobes. Her chest and abdomen CT, scanning laser ophthalmoscopy, ultrasonic cardiogram, and kidney function were normal. Her MMSE, MoCA, and SARA scores were 14/30, 13/30, and 0, respectively.

Her 33-year-old mother (II:2) had infantile spasms at 12 months. This had evolved into tonic-clonic seizures 2–3 times per week. She has been walking unsteadily since she was a toddler. Physical examination found hypomelanotic macules, fibrous plaques, ungual fibromas, shagreen patches, more severe facial angiofibromas, dental enamel pits, and intraoral fibromas symptoms (Figure 1D). Moreover, bilateral cerebellar ataxia symptoms including poor balance, gait disorder, intention tremor, and dysarthria were observed. She had no TSC-associated neuropsychiatric disorders. Cranial MRI and CT scans showed cortical dysplasia, subependymal nodules, and cerebellar atrophy (Figures 2B, C). Chest CT, abdomen CT, and ultrasonic cardiogram revealed multiple pulmonary nodules, renal angiomyolipomas, multiple renal cysts, multiple sclerotic lesions of bone, atrial septal defect, right ventricular enlargement, and mild tricuspid valve regurgitation (Figure 2D). Scanning laser ophthalmoscopy and routine kidney function blood test results were normal. Her MMSE, MoCA, and SARA scores were 29/30, 29/30, and 16, respectively. No other family members manifested any abnormality. The MMSE, MoCA, and SARA scores of the proband's father (II:1) were 29/30, 29/30, and 0, respectively. The scores of her uncle (II:3) were 30/30, 30/30, and 0, respectively.

The proband and her mother were definitely diagnosed as having TSC. Oxcarbazepine and sirolimus (1 mg/d to the proband and 2 mg/d to her mother) were administered orally. After a short time, the proband ceased oxcarbazepine treatment for no reason, and her mother stopped taking sirolimus due to mild abdominal pain. Both these decisions were against advice. Monotherapy failed to improve seizure or neuropsychiatric symptoms for 1 year. Subsequently, they were visited and given combined treatment again. After 6 months of the oxcarbazepine and sirolimus treatment, their seizure frequencies had both decreased to one time every other month. Seizure intensity and duration were also reduced. At the most recent follow-up (the proband was 15 years old, and her mother was 36 years old), their seizure frequencies were still once every other month, and the proband showed improvement in memory, numeracy, language skill, and executive function. The proband's MMSE, MoCA, and SARA scores were 21/30, 19/30, and 0, respectively. Her mother's scores were 30/30, 30/30, and 16, respectively. No significant improvement was found in their dermatologic, dental, or ataxic symptoms. EEG showed a reduction in slow waves and epileptiform discharges in the proband. CT reexamination showed that there was no significant change in subependymal nodules, cerebellar atrophy, pulmonary nodules, renal angiomyolipomas, renal cysts, and sclerotic lesions of bone in patients. Detailed clinical presentations for all family members are shown in Table 1.

**Table 1:** Clinical and genetic characteristics of members in the TSC-afflicted family.

**Parameter**	**Subjects**					
	**II:2**	**III:1**	**I:1**	**I:2**	**II:1**	**II:3**
Age (years)	36	15	65	58	49	35
Sex (M = male, F = female)	F	F	M	F	M	M
Genotype^a^	N/V	N/V	N/N	N/N	N/N	N/N
Epilepsy	+	+	–	–	–	–
Cognitive deficit	–	+	–	–	–	–
Autism spectrum disorder	–	–	–	–	–	–
Attention deficit hyperactivity disorder	–	+	–	–	–	–
Ataxia	+	–	–	–	–	–
Facial angiofibromas (*n* ≥3)	++	+	–	–	–	–
Fibrous plaques	+	+	–	–	–	–
Dental enamel pits	++	+	–	–	–	–
Intraoral fibromas	++	+	–	–	–	–
Shagreen patches	+	+	–	–	–	–
Hypomelanotic macules (*n* ≥ 3, ≥5 mm)	+	+	–	–	–	–
Ungual fibromas (*n* ≥ 2)	+	–	–	–	–	–
Cortical dysplasia	+	+	/	/	/	/
Subependymal nodules	+	+	/	/	/	/
Cerebellar atrophy	+	–	/	/	/	/
Ophthalmologic features	–	–	/	/	/	/
Cardiac structural abnormality	+	–	/	/	/	/
Multiple pulmonary nodules	+	–	/	/	/	/
Renal angiomyolipomas	+	–	/	/	/	/
Multiple renal cysts	+	–	/	/	/	/
Abnormal renal function	–	–	/	/	/	/
MMSE score	29/30	14/30	/	/	29/30	30/30
MoCA score	29/30	13/30	/	/	29/30	30/30
SARA score	16	0	/	/	0	0
Recent MMSE score	30/30	21/30	/	/	/	/
Recent MoCA score	30/30	19/30	/	/	/	/
Recent SARA score	16	0	/	/	/	/

–, normal; +, abnormal; ++, severely abnormal; /, no access.

^*a*^N, normal allele; V, allele with *TSC2* c.2742+5G>A variant.

### 3.2. Variant analysis

**Figure 3:**
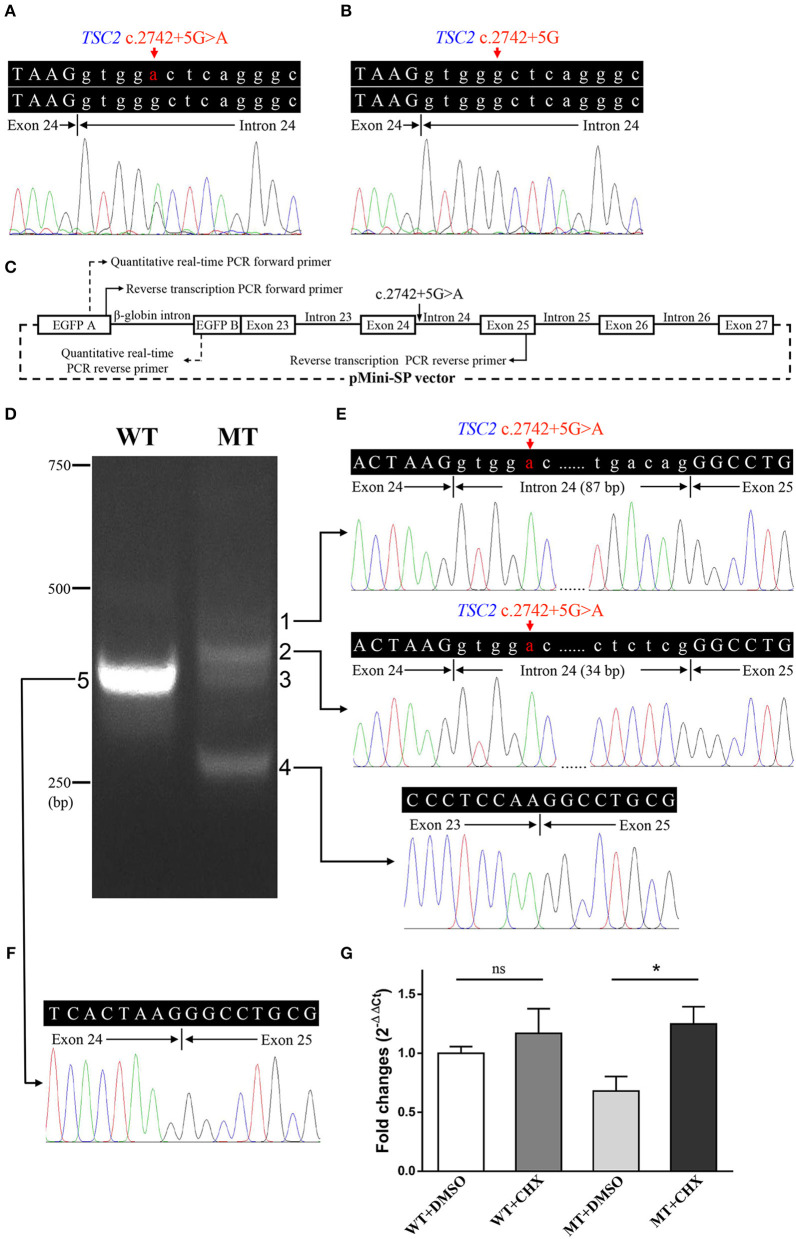
Genetic analyses and minigene assay results of the *TSC2* gene c.2742+5G>A variant. **(A)** Genomic DNA sequencing of heterozygous *TSC2* c.2742+5G>A variant in the proband. **(B)** Genomic DNA sequencing of wild-type *TSC2* gene in an unaffected member (II:1). **(C)** Minigene plasmids construction and primer sites. **(D)** The electrophoretic figure of the amplification products of cells expressing wild-type (WT) and mutant (MT) minigene plasmids. **(E)** Sequencing chromatogram of the amplification product of cells expressing mutant plasmid. Exonic sequences are shown in upper case, and intronic sequences are shown in lower case. **(F)** Sequencing chromatogram of the amplification product of cells expressing wild-type plasmid. **(G)** The relative levels of mRNA of wild-type (WT) and mutant (MT) minigene plasmids after dimethyl sulfoxide (DMSO) or cycloheximide (CHX) treatment (ns: not significant, *P < 0.001).

Whole exome sequencing detected 104,191 SNVs and 18,412 indels on the proband's gDNA. The average exome sequencing depth on target was 287.19. After filtering, 648 SNVs and 243 indels were considered as candidates. Only a candidate variant (c.2742+5G>A, rs397515076) was found in a heterozygous state in the *TSC2* gene, and no candidate variant was detected in the *TSC1* gene or other infantile epilepsy-related genes. This variant did not appear in the Genome Aggregation Database or in the in-house exome database of BGI. The RegRNA, NetGene2, NNSPLICE, ASSP, MaxEntScan, and HSF all predicted that it probably affected splicing or decreased evaluation scores by more than 50%. Six splice site prediction tools found a total of 21 cryptic donor sites in its adjacent exon and intron, including a splice site that leads to the insertion of only five amino acids (Supplementary Table 2). Sanger sequencing confirmed the presence of this variant in both the proband and her mother and its absence in other family members (Figures 3A, B). There was no significant difference in the electropherogram peak height for the c.2742+5G>A variant allele between the proband and her mother (*P* = 0.247). No variant peak was detected in the proband's grandparents. This variant has been reported in sporadic TSC cases and recorded in the Leiden Open Variation Database (Sancak et al., 2005). It was absent in the ClinVar and Human Gene Mutation Database (public version). Before this study, there had been no evidence that included segregation information or splicing data to clarify its pathogenicity according to the American College of Medical Genetics and Genomics (ACMG) guidelines for variants interpretation (Richards et al., 2015).

### 3.3. Minigene assay

The minigene assay was performed to determine the pathogenicity of this variant and to compare the accuracy of prediction tools (Figure 3C). The agarose gel electrophoresis result is shown in Figure 3D. The wild-type minigene plasmid transcription product had only one band with the expected size (363 bp) and sequence (Figures 3D, F). The cells expressing c.2742+5G>A showed four additional bands, and the wild-type band is absent (Figure 3D). Gel DNA extraction and sequencing results showed that band 1 (size of 450 bp) and band 2 (size of 397 bp) contained partial intron 24, and band 4 (size of 260 bp) lost whole exon 24 (Figure 3E). The sequencing result of band 3 indicated heterozygous peaks composed of band 2 and band 4 sequences (Supplementary Figure 1). The results of the minigene assay suggest the use of a cryptic non-canonical splice site. Compared with DMSO treatment, CHX treatment significantly increased the relative level of mutant minigene plasmid mRNA (*P* < 0.001), while no significant difference was found between CHX treatment and DMSO treatment in cells expressing wild-type minigene plasmid (*P* = 0.110, Figure 3G). The specificity of quantitative real-time PCR primer pairs was confirmed by the melting curve analysis and agarose gel electrophoresis (Supplementary Figure 2).

### 3.4. Lymphocyte mRNA analysis

**Figure 4:**
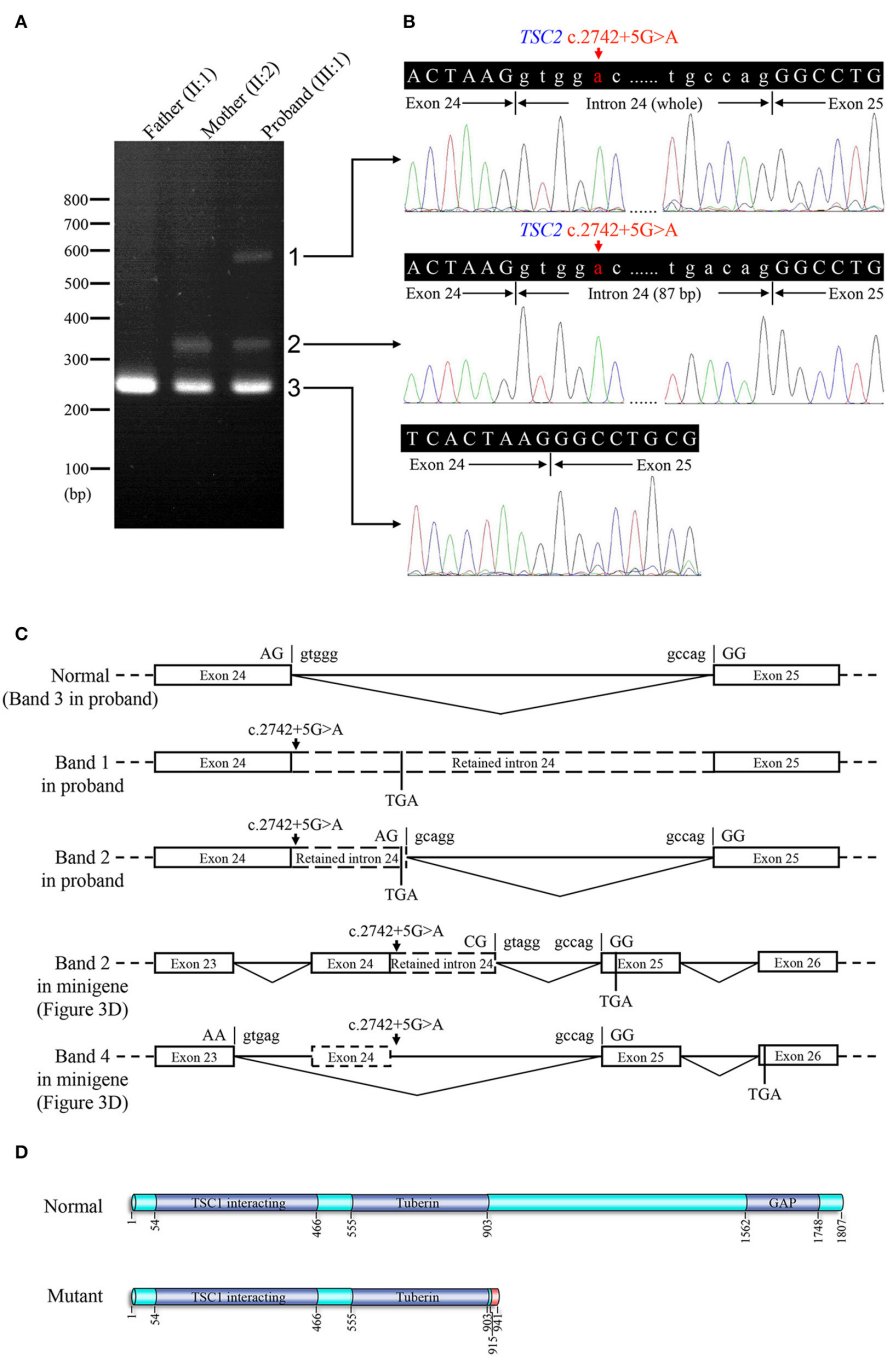
Reverse transcription PCR and sequencing results of mRNA extracted from family members in the TSC-afflicted family. **(A)** The electrophoretic figure of the amplification products of *TSC2* cDNA from unaffected and affected family members. **(B)** Sequencing chromatogram of the amplification products of *TSC2* cDNA. **(C)** A schematic diagram of the normal and mutant splicing modes of the *TSC2* gene. Splice site sequences and a termination codon (TGA) are shown, in which exonic sequences are in upper case, and intronic sequences are in lower case. **(D)** Normal and mutant tuberin proteins translated from mRNA sequences detected in patients. Functional domains are in dark blue, and the incorrect amino acid sequence is in red.

**Figure 5:**
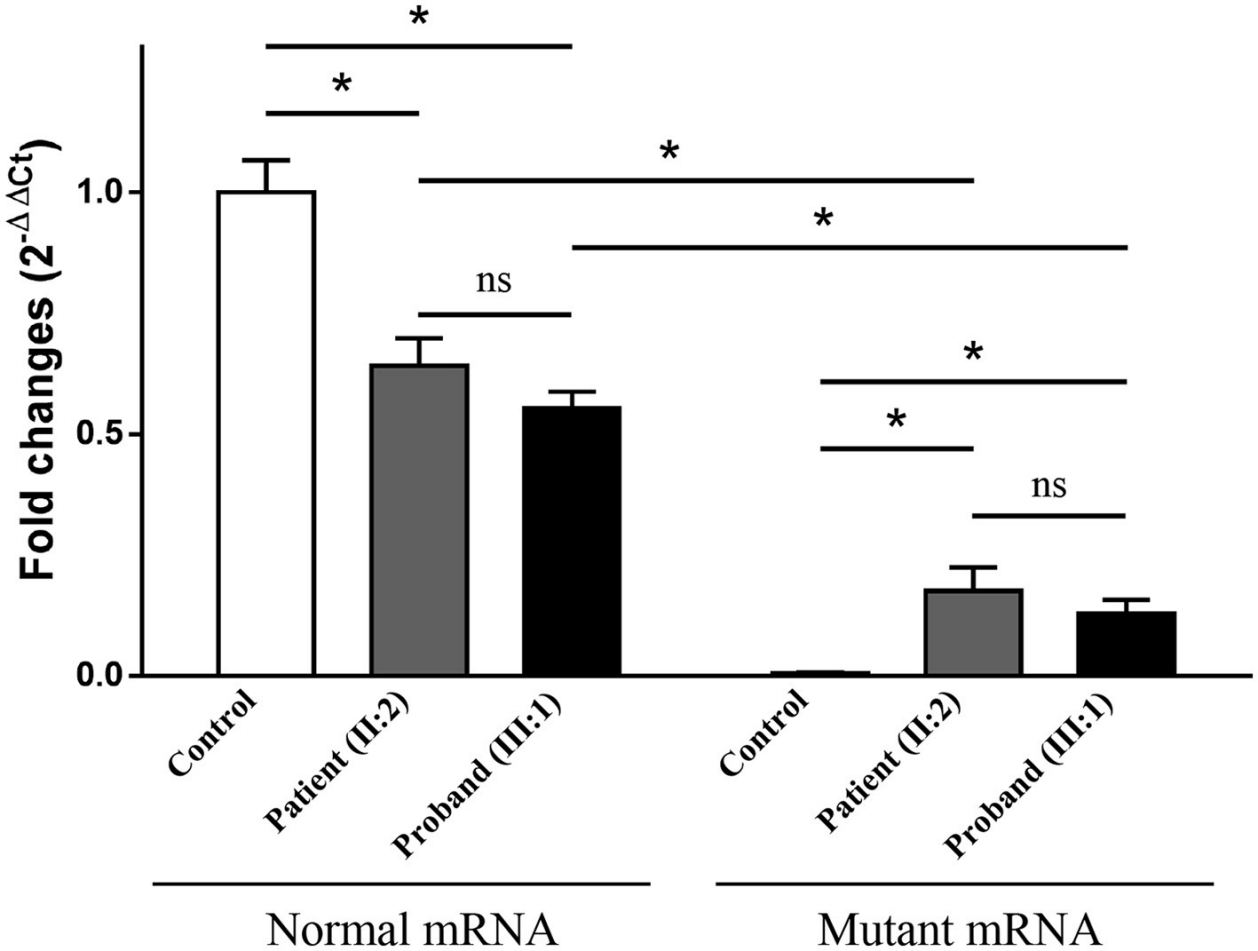
The relative levels of mRNA of the *TSC2* gene, normalized to the average normal mRNA level of controls (ns: not significant, *P < 0.001).

Electrophoresis result (Figure 4A) displayed only one band (size of 245 bp) in the PCR product of her father (II:1), two additional bands (size of 332 and 565 bp) in the proband (III:1), and one additional band (size of 332 bp) in her mother (II:2). Sanger sequencing (Figure 4B) revealed the whole retention of intron 24 in the amplification product of 565 bp (band 1), partial retention of 87 bp at the 5′ end of intron 24 in the amplification product of 332 bp (band 2), and normal splicing in the amplification product of 245 bp (band 3). The single peak at the variant allele confirmed that these intron retentions are pathological splicing changes. Both splicing alterations lead to a frame shift and premature termination (p.Gly915Valfs^*^28, Figures 4C, D). Quantitative real-time PCR revealed significant reductions in relative levels of normal *TSC2* mRNA in her affected mother (II:2, 35.89%, *P* < 0.001) and the proband (III:1, 44.64%, *P* < 0.001) compared to the controls (Figure 5). Quantitative real-time PCR against mutant *TSC2* mRNA sequence detected intron 24-retaining mRNA in two patients, and only very few non-specific amplifications were observed in controls (*P* < 0.001). The relative levels of normal *TSC2* mRNA were higher than those of mutant *TSC2* mRNA in both patients (*P* < 0.001). No significant difference was observed in the relative levels of wild-type (*P* = 0.407) and mutant (*P* = 1.000) *TSC2* mRNA between the proband and her mother. The melting curve analysis and agarose gel electrophoresis showed the specificity of quantitative real-time PCR products in Supplementary Figure 3. According to the ACMG guidelines for variants interpretation, the c.2742+5G>A variant was classified as “pathogenic” (PS2 + PS3 + PM2 + PP1 + PP3 + PP4) (Richards et al., 2015).

## 4. Discussion

Tuberous sclerosis complex is a serious inherited disease with heterogeneous manifestations, including the brain, skin, heart, and other organ abnormalities (Henske et al., 2016). Symptoms of brain involvement like epilepsy and intellectual disability are the most important factors impacting TSC-afflicted patients' quality of life (Sahin et al., 2016). This study reports a proband and her mother, both with TSC. They both carry the *TSC2* c.2742+5G>A variant, and no somatic mosaicism was observed in the proband's immediate relatives. It indicates that this variant probably surfaced *de novo* in the mother, but the possibility of germinal mosaicism and low-level somatic mosaicism cannot be excluded in the proband's grandparents. Two patients' primary clinical manifestations are epilepsy, and other symptoms vary. The proband has TSC-associated neuropsychiatric disorders, and her affected mother has more severe dermatologic features and more extensive organ involvements including the cerebellum, heart, lung, and kidney. The literature notes that only a small percentage of TSC patients have cerebellar atrophy, and cerebellar ataxia is even rarer (Jurkiewicz et al., 2006; Ertan et al., 2010). In this study, the proband's mother had typical bilateral cerebellar ataxia symptoms probably due to her severe cerebellar atrophy. Clinical manifestation variability may be partly due to the randomness of the second-hit somatic variant, which is not necessary for hamartomatous cerebral lesions but is often found in other types of hamartomas formed in TSC patients (Jozwiak and Jozwiak, 2007; Jentarra et al., 2011; Tyburczy et al., 2015). In addition, genetic milieu, epigenetic, and environmental factors cannot be excluded. A follow-up of 3 years indicated that combined oral treatment of oxcarbazepine and sirolimus, two drugs that are more accessible in developing countries, is possibly effective for TSC-related epilepsy in these two patients though their effects of monotherapy were not obvious. Previous studies showed MMSE scores reached a plateau at 10 years of age (Ouvrier et al., 1993; Rubial-Alvarez et al., 2007). The increase in MMSE and MoCA scores of the proband indicated a cognitive improvement although the influence of age could not be excluded. It suggests that a long-term placebo-controlled study with large samples for mTOR inhibitors would be valuable though a negative result has been reported in a short-term everolimus administration (Krueger et al., 2017). This improvement may also be due to the effective control of seizures. Recent studies have reported the association between seizures and neurodevelopment in infants with TSC, but the relationship between seizures and cognitive function in adolescents with TSC is not clear (Capal et al., 2017; De Ridder et al., 2021). More detailed cognitive assessment, regular EEG examination, drug concentration detection, and longer follow-up time are also necessary to understand cognitive change.

Segregation analysis is impracticable for two-thirds of patients having TSC because they are sporadic (Curatolo et al., 2008). Analyzing the functional effects of splicing variants and verifying cryptic splice sites are difficult in the RNA extracted from patients due to nonsense-mediated decay (Abramowicz and Gos, 2018). Although *in silico* prediction and minigene assay are two alternative possibilities, the pathogenicity of many intron variants which may affect splicing has not yet been determined (Tyburczy et al., 2015). We provide detailed data from heterozygous patients, including mutant mRNA sequence containing partial or whole intron 24 and a significant reduction of normal mRNA level to confirm that the *TSC2* c.2742+5G>A variant destroys the tuberin protein's structure and function. Adding CHX led to a significant increase in mutant mRNA in the minigene assay. This result and the lower level of mutant mRNA in patients' lymphocytes suggested the possibility of nonsense-mediated mRNA decay, but further experimental verification is needed. The function loss of the *TSC2* gene leads to aberrant mTOR pathway activation and multiple tumors including subependymal giant cell astrocytomas, angiomyolipomas, lymphangioleiomyomatosis, and angiofibromas (Henske et al., 2016). A pathological giant cell without normal tuberin protein is the hallmark of cerebral dysplastic lesions, and abnormal synthesis of synaptic proteins due to hyperactivity of the mTOR pathway may be the cause of epilepsy, ADHD, and ASD (Mizuguchi et al., 2021). The effect of the loss-of-function variant in the *TSC2* gene on tumorigenesis and brain function was also confirmed in animal experiments (Du et al., 2018; Mizuguchi et al., 2021). A non-canonical splice site and individual differences in the mutant mRNA sequence suggest potential defects of *in silico* prediction and minigene assay in verifying cryptic splice sites. The RegRNA, NetGene2, NNSPLICE, and ASSP failed to find the non-canonical splice site, and the MaxEntScan and HSF only gave a low score. Our findings showed that the minigene assay result may be different from *in vivo* splicing result and probably cannot reveal splicing change differences between carriers with the same splicing variant. This uncommon splicing change may enhance the understanding of splicing variants, non-canonical splice sites, and alternative splicing.

## 5. Conclusion

We identified a *TSC2* c.2742+5G>A variant as the genetic cause of a Han-Chinese family with TSC. The patients' mRNA sequencing and quantitative data first confirm its pathogenicity and suggest potential defects of *in silico* prediction and minigene assay. This variant inactivated a donor splice site and led to the use of a cryptic non-canonical alternative splice site. Detailed and heterogeneous clinical manifestations including rare cerebellar ataxia are described, and a 3-year follow-up suggests the effect of the treatment which combined anti-epilepsy drugs and sirolimus on TSC-related epilepsy and cognitive deficits. These results should improve TSC diagnosis and treatment and may contribute to a better understanding of the TSC genetic basis and splicing mechanism.

## Data availability statement

The datasets presented in this article are not readily available because the data information is in a controlled state due to the national legislation, specifically the Administrative Regulations on the Management of Human Genetic Resources of the People's Republic of China. Data of this project can be accessed after an approval application to the China National GeneBank DataBase (CNGBdb). Please refer to https://db.cngb.org/, or email: CNGBdb@cngb.org for detailed application guidance. The project accession code CNP0003735 should be included in the application.

## Ethics statement

The studies involving human participants were reviewed and approved by the Institutional Review Board of the Third Xiangya Hospital of Central South University. Written informed consent to participate in this study was provided by the participants, and minor's legal guardian/next of kin. Written informed consent was obtained from the individual(s), and minor(s)' legal guardian/next of kin, for the publication of any potentially identifiable images or data included in this article.

## Author contributions

LY and HD: conceived and designed the experiments. KF, ZS, and WZ: collected the information. KF, YG, XH, and LG: performed the experiments. KF and YG: analyzed the data. KF, LY, and HD: wrote the paper. All authors contributed to the article and approved the submitted version.

## References

[B1] Abramowicz A. Gos M. (2018). Splicing mutations in human genetic disorders: examples, detection, and confirmation. J. Appl. Genet. 59, 253–268. 10.1007/s13353-018-0444-7 29680930 PMC6060985

[B2] American Psychiatric Association. (2013). Diagnostic and Statistical Manual of Mental Disorders, 5th Edn. Washington, DC: American Psychiatric Association Publishing. 10.1176/appi.books.9780890425596

[B3] Capal J. K. Bernardino-Cuesta B. Horn P. S. Murray D. Byars A. W. Bing N. M.. (2017). Influence of seizures on early development in tuberous sclerosis complex. Epilepsy Behav. 70(Pt A), 245–252. 10.1016/j.yebeh.2017.02.007 28457992 PMC5497719

[B4] Chang T. H. Huang H. Y. Hsu J. B. Weng S. L. Horng J. T. Huang H. D. (2013). An enhanced computational platform for investigating the roles of regulatory RNA and for identifying functional RNA motifs. BMC Bioinformatics 14(Suppl. 2), S4. 10.1186/1471-2105-14-S2-S4 23369107 PMC3549854

[B5] Chung C. W. T. Lawson J. A. Sarkozy V. Riney K. Wargon O. Shand A. W.. (2017). Early detection of tuberous sclerosis complex: an opportunity for improved neurodevelopmental outcome. Pediatr. Neurol. 76, 20–26. 10.1016/j.pediatrneurol.2017.05.014 28811058

[B6] Curatolo P. Bombardieri R. Jozwiak S. (2008). Tuberous sclerosis. Lancet 372, 657–668. 10.1016/S0140-6736(08)61279-9 18722871

[B7] Curatolo P. Moavero R. de Vries P. J. (2015). Neurological and neuropsychiatric aspects of tuberous sclerosis complex. Lancet Neurol. 14, 733–745. 10.1016/S1474-4422(15)00069-1 26067126

[B8] De Ridder J. Verhelle B. Vervisch J. Lemmens K. Kotulska K. Moavero R.. (2021). Early epileptiform EEG activity in infants with tuberous sclerosis complex predicts epilepsy and neurodevelopmental outcomes. Epilepsia 62, 1208–1219. 10.1111/epi.16892 33778971

[B9] de Vries P. J. Belousova E. Benedik M. P. Carter T. Cottin V. Curatolo P.. (2018). TSC-associated neuropsychiatric disorders (TAND): findings from the TOSCA natural history study. Orphanet J. Rare Dis. 13, 157. 10.1186/s13023-018-0901-8 30201051 PMC6131901

[B10] Desmet F. O. Hamroun D. Lalande M. Collod-Béroud G. Claustres M. Béroud C. (2009). Human Splicing Finder: an online bioinformatics tool to predict splicing signals. Nucleic Acids Res. 37, e67. 10.1093/nar/gkp215 19339519 PMC2685110

[B11] Du H. Dreier J. R. Zarei M. Wu C. L. Bronson R. W. Kwiatkowski D. J. (2018). A novel mouse model of hemangiopericytoma due to loss of Tsc2. Hum. Mol. Genet. 27, 4169–4175. 10.1093/hmg/ddy289 30124871 PMC6276833

[B12] Ebrahimi-Fakhari D. Mann L. L. Poryo M. Graf N. von Kries R. Heinrich B.. (2018). Incidence of tuberous sclerosis and age at first diagnosis: new data and emerging trends from a national, prospective surveillance study. Orphanet J. Rare Dis. 13, 117. 10.1186/s13023-018-0870-y 30016967 PMC6050673

[B13] Eng L. Coutinho G. Nahas S. Yeo G. Tanouye R. Babaei M.. (2004). Nonclassical splicing mutations in the coding and noncoding regions of the ATM gene: maximum entropy estimates of splice junction strengths. Hum. Mutat. 23, 67–76. 10.1002/humu.10295 14695534

[B14] Ertan G. Arulrajah S. Tekes A. Jordan L. Huisman T. A. (2010). Cerebellar abnormality in children and young adults with tuberous sclerosis complex: MR and diffusion weighted imaging findings. J. Neuroradiol. 37, 231–238. 10.1016/j.neurad.2009.12.006 20381146

[B15] Fan K. Zhu H. Xu H. Mao P. Yuan L. Deng H. (2019). The identification of a transthyretin variant p.D38G in a Chinese family with early-onset leptomeningeal amyloidosis. J. Neurol. 266, 232–241. 10.1007/s00415-018-9125-z 30470998

[B16] Hebsgaard S. M. Korning P. G. Tolstrup N. Engelbrecht J. Rouzé P Brunak S. (1996). Splice site prediction in *Arabidopsis thaliana* pre-mRNA by combining local and global sequence information. Nucleic Acids Res. 24, 3439–3452. 10.1093/nar/24.17.3439 8811101 PMC146109

[B17] Henske E. P. Józwiak S. Kingswood J. C. Sampson J. R. Thiele E. A. (2016). Tuberous sclerosis complex. Nat. Rev. Dis. Primers 2, 16035. 10.1038/nrdp.2016.35 27226234

[B18] Houdayer C. Caux-Moncoutier V. Krieger S. Barrois M. Bonnet F. Bourdon V.. (2012). Guidelines for splicing analysis in molecular diagnosis derived from a set of 327 combined *in silico*/*in vitro* studies on BRCA1 and BRCA2 variants. Hum. Mutat. 33, 1228–1238. 10.1002/humu.22101 22505045

[B19] Humphrey A. Higgins J. N. Yates J. R. Bolton P. F. (2004). Monozygotic twins with tuberous sclerosis discordant for the severity of developmental deficits. Neurology 62, 795–798. 10.1212/01.WNL.0000113745.58425.EF 15007135

[B20] Inoki K. Li Y. Xu T. Guan K. L. (2003). Rheb GTPase is a direct target of TSC2 GAP activity and regulates mTOR signaling. Genes Dev. 17, 1829–1834. 10.1101/gad.1110003 12869586 PMC196227

[B21] Jentarra G. M. Rice S. G. Olfers S. Saffen D. Narayanan V. (2011). Evidence for population variation in TSC1 and TSC2 gene expression. BMC Med. Genet. 12, 29. 10.1186/1471-2350-12-29 21345208 PMC3051885

[B22] Jozwiak J. Jozwiak S. (2007). Giant cells: contradiction to two-hit model of tuber formation? Cell. Mol. Neurobiol. 27, 251–261. 10.1007/s10571-006-9106-0 16897363 PMC11517137

[B23] Jurkiewicz E. Józwiak S. Bekiesińska-Figatowska M. Pakieła-Domańska D. Pakuła-Kościesza I. Walecki J. (2006). Cerebellar lesions in children with tuberous sclerosis complex. Neuroradiol. J. 19, 577–582. 10.1177/197140090601900503 24351257

[B24] Krueger D. A. Sadhwani A. Byars A. W. de Vries P. J. Franz D. N. Whittemore V. H.. (2017). Everolimus for treatment of tuberous sclerosis complex-associated neuropsychiatric disorders. Ann. Clin. Transl. Neurol. 4, 877–887. 10.1002/acn3.494 29296616 PMC5740257

[B25] Mizuguchi M. Ohsawa M. Kashii H. Sato A. (2021). Brain symptoms of tuberous sclerosis complex: pathogenesis and treatment. Int. J. Mol. Sci. 22, 6677. 10.3390/ijms22136677 34206526 PMC8268912

[B26] Northrup H. Krueger D. A. International Tuberous Sclerosis Complex Consensus Group. (2013). Tuberous sclerosis complex diagnostic criteria update: recommendations of the 2012 International Tuberous Sclerosis Complex Consensus Conference. Pediatr. Neurol. 49, 243–254. 10.1016/j.pediatrneurol.2013.08.001 24053982 PMC4080684

[B27] Osborne J. P. Fryer A. Webb D. (1991). Epidemiology of tuberous sclerosis. Ann. N. Y. Acad. Sci. 615, 125–127. 10.1111/j.1749-6632.1991.tb37754.x 2039137

[B28] Ouvrier R. A. Goldsmith R. F. Ouvrier S. Williams I. C. (1993). The value of the Mini-Mental State Examination in childhood: a preliminary study. J. Child Neurol. 8, 145–148. 10.1177/088307389300800206 8505476

[B29] Reese M. G. Eeckman F. H. Kulp D. Haussler D. (1997). Improved splice site detection in Genie. J. Comput. Biol. 4, 311–323. 10.1089/cmb.1997.4.311 9278062

[B30] Richards S. Aziz N. Bale S. Bick D. Das S. Gastier-Foster J.. (2015). Standards and guidelines for the interpretation of sequence variants: a joint consensus recommendation of the American College of Medical Genetics and Genomics and the Association for Molecular Pathology. Genet. Med. 17, 405–424. 10.1038/gim.2015.30 25741868 PMC4544753

[B31] Roach E. S. (2016). Applying the lessons of tuberous sclerosis: the 2015 Hower Award Lecture. Pediatr. Neurol. 63, 6–22. 10.1016/j.pediatrneurol.2016.07.003 27543366

[B32] Rubial-Alvarez S. Machado M. C. Sintas E. de Sola S. Böhm P. Peña-Casanova J. (2007). A preliminary study of the mini-mental state examination in a Spanish child population. J. Child Neurol. 22, 1269–1273. 10.1177/0883073807307098 18006955

[B33] Sahin M. Henske E. P. Manning B. D. Ess K. C. Bissler J. J. Klann E.. (2016). Advances and future directions for tuberous sclerosis complex research: recommendations from the 2015 strategic planning conference. Pediatr. Neurol. 60, 1–12. 10.1016/j.pediatrneurol.2016.03.015 27267556 PMC4921275

[B34] Sancak O. Nellist M. Goedbloed M. Elfferich P. Wouters C. Maat-Kievit A.. (2005). Mutational analysis of the TSC1 and TSC2 genes in a diagnostic setting: genotype–phenotype correlations and comparison of diagnostic DNA techniques in tuberous sclerosis complex. Eur. J. Hum. Genet. 13, 731–741. 10.1038/sj.ejhg.5201402 15798777

[B35] Tyburczy M. E. Dies K. A. Glass J. Camposano S. Chekaluk Y. Thorner A. R.. (2015). Mosaic and intronic mutations in TSC1/TSC2 explain the majority of TSC patients with no mutation identified by conventional testing. PLoS Genet. 11, e1005637. 10.1371/journal.pgen.1005637 26540169 PMC4634999

[B36] Wang M. Marín A. (2006). Characterization and prediction of alternative splice sites. Gene 366, 219–227. 10.1016/j.gene.2005.07.015 16226402

[B37] Yuan M. Guo Y. Xia H. Xu H. Deng H. Yuan L. (2021). Novel SCN5A and GPD1L variants identified in two unrelated Han-Chinese patients with clinically suspected Brugada syndrome. Front. Cardiovasc. Med. 8, 758903. 10.3389/fcvm.2021.758903 34957250 PMC8692717

