# Supplementary material for: The TSC2 c.2742+5G>A variant causes variable splicing changes and clinical manifestations in a family with tuberous sclerosis complex
Source: Front Mol Neurosci. 2023 Apr 20;16:1091323. doi: 10.3389/fnmol.2023.1091323 (PMC10157042; doi:10.3389/fnmol.2023.1091323)
Supplement: Supplementary file 1 [file Data_Sheet_1.pdf]

Supplementary Material

1 Supplementary Figures and Tables

1.1 Supplementary Figures

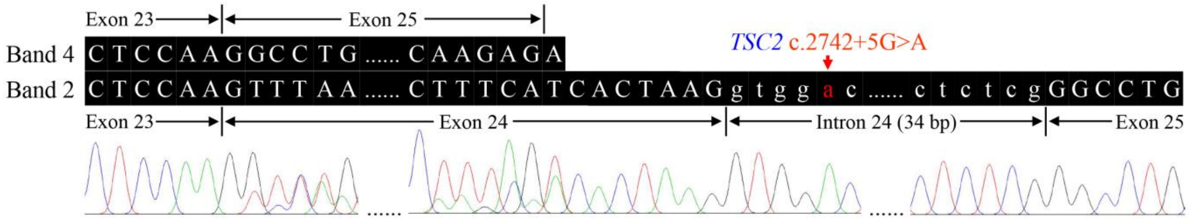

Supplementary Figure 1. Sequencing chromatogram of the band 3 in the electrophoretic figure of minigene assay (Figure 3D). Exonic sequences are shown in upper case, and intronic sequences are shown in lower case.

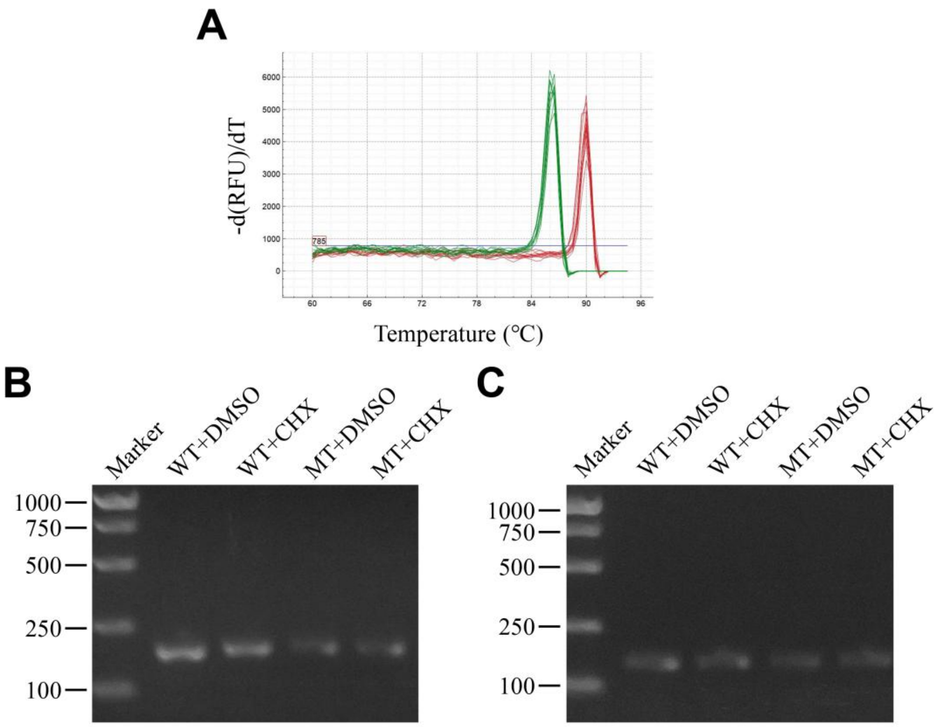

Supplementary Figure 2. Melting curve analysis and agarose gel electrophoresis of quantitative real-time PCR products in the minigene assay. **(A)** Melt peak of the quantitative real-time PCR products of minigene sequence (red) and the actin beta gene (green). **(B)** The electrophoretic figure of the quantitative real-time PCR products of minigene sequence. **(C)** The electrophoretic figure of the quantitative real-time PCR products of the actin beta gene. WT: cells expressing wild-type minigene plasmid; MT: cells expressing mutant minigene plasmid; DMSO: dimethyl sulfoxide treatment; CHX: cycloheximide treatment.

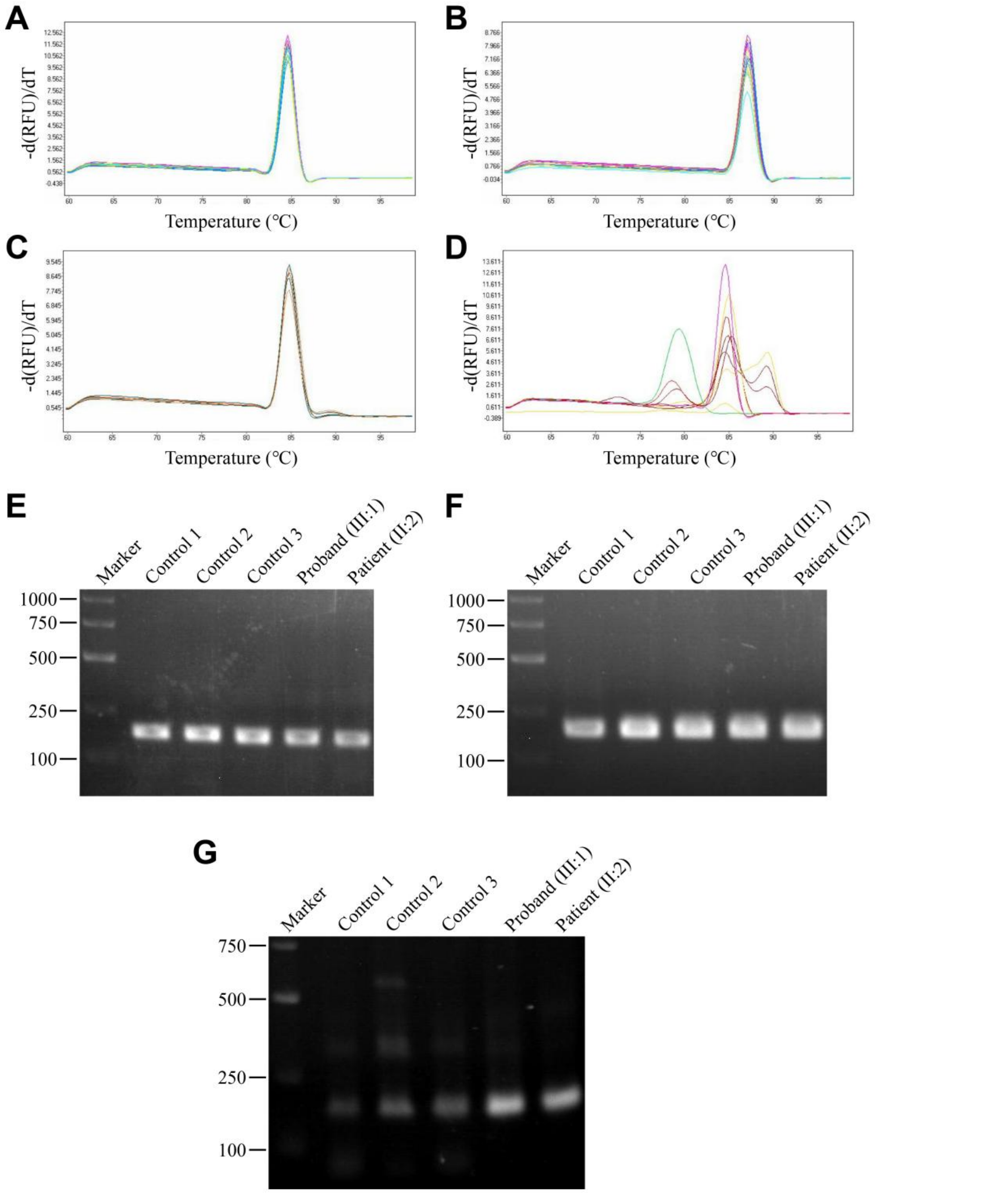

Supplementary Figure 3. Melting curve analysis and agarose gel electrophoresis of quantitative real-time PCR products in the lymphocyte mRNA analysis. **(A)** Melt peak of the quantitative real-time PCR products of the actin beta gene. **(B)** Melt peak of the quantitative real-time PCR products of wild-type *TSC2* cDNA. **(C)** Melt peak of the quantitative real-time PCR products of mutant *TSC2* cDNA in TSC patients. **(D)** Melt peak of the quantitative real-time PCR products of mutant *TSC2* cDNA in controls. **(E)** The electrophoretic figure of the quantitative real-time PCR products of the actin beta gene. **(F)** The electrophoretic figure of the quantitative real-time PCR products of wild-type *TSC2* cDNA. **(G)** The electrophoretic figure of the quantitative real-time PCR products of mutant *TSC2* cDNA.

1.2 Supplementary Tables

Supplementary Table 1 List of primers used in this study

Primer	Sequence	Amplicon size (bp)	Application
gTSC2_IVS23_F	5’-CCCCCTTCTCATCTCAGGTT-3’	217	Detecting *TSC2* c.2742+5G>A variant in genomic DNA
gTSC2_IVS24_R	5’-CTCCACCTGCCTGTCACTCT-3’		
gTSC2_EX23_F[a]	5’-AAGCTTGGTACCGAGCTCGGATCCACTCTGGCCAGGCTGCCGCACCTCTACAG-3’	822	Amplifying the front part of minigene region from genomic DNA
gTSC2_EX25_R	5’-CAGGCCGTACCTCTTGGGTCTCTCGTTGAGACTAGTA-3’		
gTSC2_EX25_F	5’-GACCCAAGAGGTACGGCCTGCGGGGGTGTGCCTGGAGT-3’	2645	Amplifying the latter part of minigene region from genomic DNA
gTSC2_EX27_R[a]	5’-TTAAACGGGCCCTCTAGACTCGAGCTCTTCGGGACAGCCGTGAAGTTGGAGA-3’		
cMiniRT_F	5’-GGCTAACTAGAGAACCCACTGCTTA-3’	363	Detecting splicing changes in minigene assay
cTSC2_EX25_R	5’-CTCTTGGGTCTCTCGTTGAGACTA-3’		
pMiniSP-EGFP-F	5’-CTTCAAGATCCGCCACAACA-3’	183	Quantitative real-time PCR primers for minigene RNA sequence
pMiniSP-EGFP-R	5’-GTCACGAACTCCAGCAGCA-3’		
qACTB_F1	5’-CCTGGCACCCAGCACAAT-3’	144	Quantitative real-time PCR control gene (the actin beta gene) primers in minigene assay
qACTB_R1	5’-GGGCCGGACTCGTCATAC-3’		
cTSC2_EX23_F	5’-CCGCACCTCTACAGGAACTT-3’	245	Detecting splicing changes between exons 23 and 25 of *TSC2* gene in complementary DNA
cTSC2_EX25_R2	5’-GCCCTGAAGCTGTCCTTCTC-3’		

qTSC2_EX23_FW<br>qTSC2_EX25_RW	5’-CAGTATGCCAGTGTGTTCGCCATCTC-3’<br>5’-ACATTGGACCGCAGGCCCTTAGT-3’	170	Quantitative real-time PCR primers for the *TSC2* wild-type messenger RNA sequence
qTSC2_EX23_FM<br>qTSC2_IVS24_RM	5’-TGTTCGCCATCTCCCTGCCGTA-3’<br>5’-GCCTACCGAGAGACACAGCCTTCA-3’	180	Quantitative real-time PCR primers for the *TSC2* mutant messenger RNA sequence
qACTB_F2<br>qACTB_R2	5’-CTGGCACCACACCTTCTACA-3’<br>5’-AGAGGCGTACAGGGATAGCA-3’	181	Quantitative real-time PCR control gene (the actin beta gene) primers in lymphocyte messenger RNA analysis

[a] The underlined sequences are used for plasmid construction.

Supplementary Table 2 Cryptic donor sites in *TSC2* gene exon 24 and intron 24 analyzed by *in silico* tools, minigene assay, and mRNA sequencing

Location	Sequence[a]	RegRNA minimum free energy	NetGene2 confidence	NNSPLICE score	ASSP score	MaxEntScan score[b]	HSF matrix value	Minigene assay	Patients' mRNA sequencing
c.2653_2661	ATCgtgtgt	-	-	-	-	-	72.91	-	-
c.2657_2665	TGTgtctgg	-	-	-	-	-	65.87	-	-
c.2671_2679	CACgtcata	-	-	-	-	-	70.25	-	-
c.2682_2690	CATgtggtt	-	-	-	-	-	69.74	-	-
c.2685_2693	GTGgttcat	-	-	-	-	-	69.65	-	-
c.2694_2702	CAGgtgccg	-	-	-	9.408	6.46	76.82	-	-
c.2742+13_2742+21	CCGgtgaag	-	0.55	-	-	4.01	78.83	-	-
c.2742+32_2742+40	TCGgtaggc	-24.60	0.55	0.68	8.542	7.75	82.2	Alternative splicing	-
c.2742+68_2742+76	ATGgtcggg	-	-	-	-	-	78.09	-	-
c.2742+78_2742+86	AGAgtgaca	-	-	-	-	-	68.15	-	-
c.2742+85_2742+93	CAGgcaggt	-	-	-	-	2.53	65.66	Alternative splicing	Alternative splicing
c.2742+89_2742+97	CAGgtggag	-	-	-	5.084	2.57	77.8	-	-
c.2742+95_2742+103	GAGggcagt	-	-	-	-	-	65.53	-	-
c.2742+99_2742+107	GCAgtggga	-	-	-	-	-	69.92	-	-
c.2742+107_2742+115	AGGgtgttt	-	-	-	-	-	74.02	-	-
c.2742+109_2742+117	GGTgtttgg	-	-	-	-	-	66.29	-	-
c.2742+186_2742+194	GGGgtggga	-	-	-	8.239	-	80.07	-	-
c.2742+197_2742+205	TGGgtgccg	-	-	-	-	-	69.22	-	-
c.2742+259_2742+267	CTGgtgagg	-	0.47	0.91	14.919	8.30	88.8	-	-
c.2742+294_2742+302	ACTgtctgg	-	-	-	-	-	67.17	-	-
c.2742+300_2742+308	TGGgtgtgc	-	-	-	7.162	2.34	79.43	-	-

mRNA, messenger RNA; NNSPLICE, Splice Site Prediction by Neural Network; ASSP, Alternative Splice Site Predictor; MaxEntScan, Maximum Entropy Scan; HSF, Human Splicing Finder.

[a] Predicted exonic sequences are shown in upper case, and predicted intronic sequences are shown in lower case.

[b] Only MaxEntScan score more than 2.00 is shown.
